# Stabilizing Techniques and Guided Imagery for Traumatized Male Refugees in a German State Registration and Reception Center: A Qualitative Study on a Psychotherapeutic Group Intervention

**DOI:** 10.3390/jcm8060894

**Published:** 2019-06-22

**Authors:** Catharina Zehetmair, Inga Tegeler, Claudia Kaufmann, Anne Klippel, Luise Reddemann, Florian Junne, Sabine C. Herpertz, Hans-Christoph Friederich, Christoph Nikendei

**Affiliations:** 1 Center for Psychosocial Medicine, Department for General Internal Medicine and Psychosomatics, University Hospital of Heidelberg, 69115 Heidelberg, Germany; Inga.Tegeler@med.uni-heidelberg.de (I.T.); Claudia.Kaufmann@med.uni-heidelberg.de (C.K.); Anne.Klippel@med.uni-heidelberg.de (A.K.); Hans-Christoph.Friederich@med.uni-heidelberg.de (H.-C.F.); Christoph.Nikendei@med.uni-heidelberg.de (C.N.); 2 Center for Psychosocial Medicine, Department of General Psychiatry, University Hospital of Heidelberg, 69115 Heidelberg, Germany; sabine.herpertz@med.uni-heidelberg.de; 3 Department of Clinical Psychology, Psychotherapy and Psychoanalysis, University of Klagenfurt, 9020 Klagenfurt, Austria; l.reddemann@t-online.de; 4 Department of Psychosomatic Medicine and Psychotherapy, Medical University Hospital of Tuebingen, 72076 Tuebingen, Germany; Florian.Junne@med.uni-tuebingen.de

**Keywords:** stabilizing techniques, guided imagery, refugees, qualitative analyses, post-traumatic stress disorder, group session, body scan

## Abstract

Refugees have an increased risk of developing mental health problems. Due to the unstable setting in refugee state registration and reception centers, recommended trauma-focused treatment approaches are often not applicable. For this purpose, we devised a suitable therapeutic approach to treat traumatized refugees in a German state registration and reception center: Group therapy, focusing on stabilizing techniques and guided imagery according to Reddemann (2017). From May 2017 to April 2018, we conducted semi-structured interviews with *n* = 30 traumatized refugees to assess their experiences with the stabilizing techniques and guided imagery in group sessions and self-practice. Participants mainly reported that they had more pleasant feelings, felt increasingly relaxed, and could better handle recurrent thoughts. Additionally, the participants noticed that their psychosocial functioning had improved. The main difficulties that participants encountered were feeling stressed, having difficulties staying focused, or concentrating on the techniques. During self-practice, the participants found it most challenging that they did not have any verbal guidance, were often distracted by the surroundings in the accommodation, and had recurrent thoughts about post-migratory stressors, such as insecurity concerning the future or the application for asylum. Our results show that stabilizing techniques and guided imagery according to Reddemann (2017) are a suitable approach to treat traumatized refugees living in volatile conditions.

## 1. Introduction

Numerous studies report an elevated risk for mental health problems among refugees [1,2,3]. The most common disorders are posttraumatic stress disorder (PTSD), depression, and anxiety disorders [1,4,5]. Apart from traumatizing experiences in the home country or during the escape [2,5,6,7], post migratory stressors contribute to the onset or the aggravation of pre-existing mental health problems [6,8]. Examples for burdening factors after the arrival in their host country are the refugees’ insecurity about their application for asylum and accommodation, their limited scope of action, the loss of their particular social role, and unemployment [2,5,6]. However, despite the obvious need for psychosocial support, there is still a lack of appropriate psychosocial treatment procedures and mental health care provision [6,9].

Different therapeutic interventions have been proposed for the treatment of refugees who have symptoms of posttraumatic stress. The following approaches have been investigated in the most detail: Narrative exposure therapy (NET), trauma focused cognitive behavioral therapy (CBT), and eye movement desensitization and reprocessing (EMDR). Several studies show the effectiveness of these therapeutic interventions for the reduction of PTSD symptoms [1,3,6,10]. Further studies demonstrate that EMDR or CBT in a group setting were able to improve the PTSD symptoms of refugees and asylum seekers [11,12,13,14,15,16]. Jacobs et al. [16] introduces a collective, narrative practice as an effective way for unaccompanied refugee minors to deal with traumatic experiences. Further studies show that group therapy focusing on empowerment increases the overall feeling of well-being among traumatized refugees [17,18]. Some authors promote the use of dance, music, or art therapy to reduce PTSD symptoms [14,19,20,21]. However, despite these first promising results [3], there is still a lack of evidence—especially for treatment approaches in group settings which refrain from trauma exposure. These are of particular importance, considering that during the early stages of the application for asylum there is no guarantee for the safe and stable setting needed for trauma confrontation therapy.

According to previous research, the stabilizing techniques and guided imagery in line with Reddemann [22] turn out to be a promising treatment approach for refugees with PTSD [13,23]. So far, stabilizing techniques and guided imagery are used as one of several other possible strategies for symptom control within a multi-modular approach for unaccompanied refugee minors and for traumatized Bosnian adult refugees; in both cases, any PTSD symptoms could be reduced [13,23]. Furthermore, our research group previously implemented stabilizing techniques and guided imagery sessions for traumatized male refugees in a German state registration and reception center to offer easily accessible support. From a longitudinal pre-post study, we are able to reveal that a group concept focusing on stabilizing techniques and guided imagery according to Reddemann [22] significantly reduces perceived distress and anxiety symptoms, while inducing and strengthening pleasant feelings and the feeling of being in control [24].

Based on the study of Zehetmair et al. [24], our aim was to gain a deeper understanding of what the refugees experienced while taking part in a group therapy in which the stabilizing techniques and guided imagery according to Reddemann [22] were practiced. For this purpose, we applied a qualitative in-depth analysis. Our research questions were the following: (1) How do the participants perceive the group concept focusing on stabilizing techniques and guided imagery? (2) What do the participants achieve while practicing the techniques during the guided group session on the one hand, and during self-practice on the other hand? (3) What inhibiting factors and difficulties are experienced in group-practice and self-practice?

## 2. Material and Methods

### 2.1. Participants and Setting

The study was conducted in the refugee state registration and reception center ‘Patrick Henry Village’ (PHV) Heidelberg-Kirchheim, Germany. The PHV is the name of a former American military barracks, and currently accommodates about 1200 newly-arrived refugees [25]. Within the refugee state registration and reception center, the University Hospital of Heidelberg has set up a medical walk-in clinic which includes a psychosocial walk-in clinic with consultation hours for refugees suffering from mental illness [25,26].

In the psychosocial walk-in clinic, experienced psychotherapists and psychiatrists offer consultations including clinical diagnosis, stabilizing interventions and pharmacological treatment. Before the start of the present study, the psychotherapists and psychiatrists working in the psychosocial walk-in clinic in the state registration and reception center were informed about the structure, content, and procedure related to our group therapy offer that focuses on stabilizing techniques and guided imagery. Refugees who sought help in the psychosocial walk-in clinic [26], and met our study’s inclusion criteria, were referred to the group therapy sessions by the clinic’s psychotherapists and psychiatrists. Inclusion criteria were the diagnosis of posttraumatic stress disorder (PTSD) given by the psychotherapists and psychiatrists in the psychosocial walk-in clinic, being of male gender, and possessing the ability to speak and understand English. Exclusion criteria were addiction, current psychosis, traumatic brain injury, and an age below 18 years. In a previous publication, we presented the quantitative analysis of the effects of stabilizing techniques and guided imagery on psychometric measures [24]. In order to gather qualitative data on the stabilization group therapy, we conducted semi-structured interviews with the participants at three different stages from May 2017 to June 2018. All of the study participants had either applied for asylum in Germany before, or were in the middle of the application process during the intervention.

### 2.2. Study Design and Semi-Structured Qualitative Interviews

Based on the prospective longitudinal follow-up study by Zehetmair et al. [24], we conducted a qualitative study on group therapy sessions using stabilizing techniques and guided imagery in the refugee state registration and reception center PHV. Before the individual’s first attendance in the group session with the focus on stabilizing techniques and guided imagery, the participants were asked to complete baseline questionnaires evaluating their psychological burden. Semi-structured qualitative interviews were carried out with participants of the stabilization group to assess facilitating and inhibiting factors for practicing the stabilizing techniques and guided imagery, as well as their impression about the group offer.

In order to increase the credibility of the results, we designed semi-structured interviews based on the methodological approach by Helfferich [27]. The interview questions were developed according to the guidelines of the COREQ-Checklist [28]. The interviews comprised key questions which were followed by probing questions; for further details, some clarifying questions could be added. Table 1 shows the interview guidelines and the key questions. The interviews were conducted at three different points in time: After the first (T1) and after the fifth group therapy session (T2), and two weeks after the individual participant had attended the group session for the last time (follow-up). Both the T1 and the T2 interviews were conducted personally right after a group therapy session. The follow-up interviews were done by telephone. For the interview, each participant was met individually by PhD candidates of the University Hospital Heidelberg (CZ, IT). Both interviewers had been especially trained to conduct the interviews according to the guidelines, and were supervised by an experienced researcher familiar with qualitative research methods (CN).

### 2.3. Ethical Approval

The study was approved by the ethics committee of the University of Heidelberg (S-640/2016) and all participants gave their written informed consent in accordance with the Declaration of Helsinki.

### 2.4. Procedure of the Group Therapy Sessions with Focus on Stabilizing Techniques and Guided Imagery

The stabilizing techniques and guided imagery that constitute the focus of this study aim to provide individuals with self-regulatory strategies to remain in the ‘here and now’, to evoke feelings of safety and comfort, and to renew resources. These techniques are cornerstones of the psychodynamic imaginative trauma therapy (PITT [29]) which states that stabilization is a necessary precondition for a successful trauma processing and confrontation.

The group therapy sessions took place twice a week on Mondays and Thursdays. New group members were asked to arrive a bit earlier so that the therapists could discuss psychoeducational issues with them, and inform them of the content and structure of the group sessions. Every group therapy session followed the same pattern: (1) Short introduction round where participants were asked about their personal well-being, (2) Mindful Breathing, (3) Body Scan and (4) guided imagery technique (‘The inner safe place’ or ‘The Tree’), (5) a short discussion round on individual feelings and reactions during the therapy session [24].

### 2.5. Quantitative and Qualitative Data Analysis

For the quantitative statistical analysis, we used the Statistical Package for the Social Sciences (SPSS) program version 24 [29]. The demographic variables and baseline characteristics were analyzed using descriptive statistics (frequencies, means, and standard deviations). The interviews were digitally recorded and transcribed verbatim by independent co-workers using the guidelines for interview transcription presented in Mayring et al. [30]. The content analysis followed the principles of inductive content analysis described by Mayring [30], using the software MAXQDA [31]. For this, we first went through each transcribed interview and identified single or multiple sentences as quotes, representing the most elemental unit of meaning [32]. Accordingly, these quotes were coded and hereby labeled with a term or a short sentence (coding) to summarize them into a relevant category. Next, we checked if further coded quotes matched one of these categories, if coded quotes from the interviews had to be summarized, or if a new category needed to be established. After having so worked through about 40% of the text material, we revised the category and the coded quotes system and examined whether or not the categories, summarizations, and definitions were logical. Only then did we complete the analysis of the whole text material. Thereafter, the categories were grouped into main themes until we could define a number of relevant main themes for all participants. Finally, an inter-coder agreement check was conducted by a further independent researcher. We discussed the categories and main themes in order to reach a consensus or adjust them if necessary [30]. We summarized the statements of all interviews and highlighted noteworthy differences between the interviews.

## 3. Results

### 3.1. Sample Characteristics

At different time points during the study period, we conducted semi-structured interviews with *n* = 30 out of the 46 participants who took part in the group therapy sessions.

For the results of the quantitative evaluation, see Zehetmair et al. [24]. We conducted T1 interviews (after the first session attendance) with *n* = 16 participants, T2 interviews (after the fifth session attendance) with *n* = 9 participants, and follow-up interviews (two weeks after the last session attendance) with *n* = 26 participants. In total, only five participants (15%) were able to take part in all three interviews (T1, T2 and the follow-up). Reasons for this were a high drop-out rate between the T1 and T2 interview on the one side. Consequently, *n* = 18 out of 30 participants (60%) did not attend the fifth session; on the other hand, we stopped conducting T1 interviews before the T2 interviews (commonly termed content saturation, see [33]). In detail, the distribution was the following: Five participants (15%) took part in all three interviews, nine participants (30%) took part in interview T1 and the follow-up, and two participants (10%) took part in interview T2 and the follow-up. Two participants took part in interview T1 only, one participant took part in interview T2 only, and ten participants (33%) took part in the follow-up only.

Table 2 depicts sample characteristics for all of the participants that we interviewed for this qualitative study. Besides the clinical PTSD diagnosis, some participants of this study were diagnosed with further comorbid mental disorders by the psychiatrists and psychotherapists working in the psychosocial walk-in clinic. The most prominent comorbid mental disorders were depressive disorder (severe: *n* = 4, 13.3%; moderate: *n* = 5; 16.7%), somatoform disorder (*n* = 3, 10%), psychotic disorder (currently remitted *n* = 3, 10%), stammering (*n* = 1, 3.3%) and alcohol abuse (*n* = 1, 3.3%). Furthermore participants most frequently reported insomnia (*n* = 22, 73.3%), headaches (*n* = 15, 50.0%), back pain (*n* = 10, 33.3%), chest pain (*n* = 12, 40.0%) and stomach pain (*n* = 8, 26.7%).

The baseline scores for PTSD, depression, and anxiety disorders are presented in Table 2 as previously reported by Zehetmair et al. [24]. On average the participants reported 4.61 trauma events (*SD* = 1.77, *Range*: 1–8). The most frequent trauma events were seeing someone being killed or seriously injured (*n* = 25, 83.3%), torture (*n* = 22, 73.3%), physical assault or sexual abuse (*n* = 19, 63.3%) and imprisonment (*n* = 19, 63.3%). According to the consultation in the psychosocial walk-in clinic, all participants were in need of a psychotherapeutic therapy. 73.3% of the participants had not had any treatment due to mental health issues before. On average, the participants attended 7.03 group therapy sessions (*SD* = 8.56, range 1–40).

The follow-up interviews revealed that *n* = 23 (92%) participants stopped coming to the group therapy sessions because they had been transferred to other accommodations or municipal housing. *n* = 1 participant did not attend anymore because of physical complaints, and *n* = 2 participants ceased to take part because they did not feel comfortable with the other group members. Except for one participant, all of the participants who had been transferred (*n* = 22) to other accommodation reported that there was no comparable group offer available at their new accommodation. However, six participants stated that there was psychosocial support offered by the church or charity institutions, or that there was a doctor or a psychologist present. *n* = 14 (61%) (out of the 23 participants who had been transferred) said that they would have liked to continue participating in the group therapy sessions focusing on stabilizing techniques and guided imagery or a similar group therapy at their new accommodation.

### 3.2. Results of the Qualitative Interviews

We identified 210 single statements from the T1 and T2 interviews and 143 single statements from the follow-up interviews as quotes which were coded. From these quotes, we derived eleven categories which were then summarized into four main themes. In the following paragraphs, we will present the main themes and categories. In Table 3 we give examples of statements for each individual category within the main themes.

**(A) Motivation to attend the first group session (35 quotes)**

The participants’ statements regarding their motivation to attend the first session can be divided in internal and external motivation.

Many refugees reported that psychiatrists or psychologists from the psychosocial walk-in clinic had motivated them to join the group therapy sessions to try stabilizing techniques and guided imagery sessions [26]. Before attending for the first time, some of the participants stated that they had felt uncertain whether they should really try out this group therapy. One of the main reasons to participate in the group sessions was that many refugees hoped that it would help them to feel better or more like their ‘old selves’ again. One participant said that he had started attending the sessions because he was worried that he would lose his family due to his mental health problems.

**(B) Appraisal of the group setting (67 quotes)**

The participants made statements about their impression as well as perceived positive and negative experiences of the group setting focusing on stabilizing techniques and guided imagery. In the statements on our group setting, reference was made to the presence of other participants and the therapists, as well as the atmosphere within the group setting.

- **Other group members being present (23 quotes):** This category summarizes all of the statements participants made regarding the presence of other group members. Most of the statements referred to a normalization of the participants’ symptoms. The participants thought that their own mental distress became more ‘normal’ because there were other group members with similar problems in the group sessions. Next to normalization, exchange within the group was highly valued by the participants. During the group sessions, they found it helpful to experience peer-support and to be able to share personal experiences, i.e., practicing stabilizing techniques and guided imagery together. Several participants mentioned that they had found it especially important to encounter more experienced participants in their very first session. This enabled them to learn about the ways in which the techniques had already helped them. Even though the group setting had positive effects on the participants through normalization and exchange, uncertainties were also reported. A few participants felt uncertain, whether they could trust the other group members. One participant noted that he felt overwhelmed to be confronted with the others’ experiences, while not being able to gain the same relief from the techniques as expressed by the other participants.
- **Atmosphere of the group (25 quotes):** This category includes the spatial atmosphere as well as the atmosphere regarding the group structure. The participants considered the atmosphere of the therapy room and the instructions given by the therapists as valuable aspects. Even though some participants stated to have been nervous before the first session attendance, the atmosphere was welcoming and peaceful for them. Most of the participants appreciated that the sessions always followed the same procedure. One participant declared that he had not understood all of the instructions, while other participants stated that the instructions were formulated in a simple and comprehensible way. Another participant felt uncomfortable in the enclosed space and did not like closing his eyes.
- **Support by the therapists (19 quotes):** Statements concerning the interpersonal level between the therapists and the participants are summarized here. The interaction between the participants and the therapists provided support. Some participants pointed out that they liked the open-minded and respectful way the therapists spoke to them. One participant stated that it helped him to talk to the therapists during the group session, but that he had felt lonely as soon as he left the room. Many participants said that they felt inspired to reflect on changes and improvements in their mental state by the guidance and advice given them by the therapists. One participant appreciated that the therapists acted as ‘role models’ and participated in the stabilizing techniques and guided imagery, which made it easier for him to understand the instructions.

**(C) Achievements by practicing stabilizing techniques and guided imagery (156 quotes)**

A central aspect of the participants’ statements was that they had noticed that the stabilizing techniques and guided imagery had positive effects for them—both when practiced during the group sessions and when practiced individually. Further, they described what they positively perceived and improved during, immediately after and in the long term while practicing the stabilizing techniques and guided imagery. These experiences and positive effects can be divided in the categories emotional, physical, cognitive level and self-efficacy, which are described in the following:

- **Effects on an emotional level (61 quotes)**: This category sums up all the statements the participants made regarding effects on emotional and affective level. The participants stated that the sessions focusing on stabilizing techniques and guided imagery generally helped them to experience more pleasant emotions like calming, happiness, optimism, safety, and strength. Some participants pointed out that they felt tired after the session, or even slept during the session for some time. Furthermore, some of the participants described feeling a stronger connection with their own emotions. Interestingly, three participants noted that they felt more ‘normal’ again. Only a few participants stated that they felt less anxious or distressed.
- **Effects on a physical level (46 quotes):** Statements concerning effects and improvements on the physical level are summarized here. Frequently, participants stated that they felt a stronger connection with their bodies or generally feeling better in relation to their body. They ascribed this to having practiced the stabilizing techniques and guided imagery either in the group or individually. The participants characterized this physical wellbeing as feeling more relaxed, stronger, or ‘free’. Moreover, the participants noticed that their pain was reduced, and they could concentrate better. One participant highlighted how helpful it was for him to imagine himself in his ‘safe place’; others mentioned that the Body Scan helped them to feel better in their bodies. In the follow-up interviews, the participants also described that their sleep had improved.
- **Effects on a cognitive level (27 quotes):** This category includes effects on a cognitive level which were described due to practicing the stabilizing techniques and guided imagery. The participants noticed changes regarding their thinking patterns, such as having more positive and pleasant thoughts or being able to better deal with recurrent, negative thoughts. Furthermore, several participants described that they felt increasingly capable of focusing on the present moment, instead of thinking about the future or the past. Some participants said that the Mindful Breathing in particular helped them to stay grounded in the present. In the follow-up interviews, the participants described that they were able to use the stabilizing techniques and guided imagery to stop certain thoughts. However, in retrospect, cognitive improvements were reported less frequently.
- **Effects on self-efficacy (22 quotes):** This category summarizes the statements of the T2 interviews and follow-up interviews which described the effects on self-efficacy, whereas no statements could be detected in the T1 interviews. In the T2 interviews, several participants pointed out that they felt more competent in social interactions. One participant stated that attending the group sessions had helped him to trust other people again. In the follow-up interviews, the participants described that both their feeling of being in control and their self-confidence had increased.

**(D) Difficulties in practicing the techniques (92 quotes)**

The participants described several challenges they faced in connection with the stabilizing techniques and the guided imagery—both during group-practice and while practicing individually. These can be obstacles that prevent the participants from practicing or interfere with practicing the stabilizing techniques and guided imagery. We have divided these difficulties into three categories: Symptom-related difficulties, challenges caused by the surroundings, and lacking structure.

- **Symptom-related difficulties (39 quotes):** This category covers all difficulties and hampering obstacles related to the symptoms of the participants. During the group sessions, the main challenges faced by the participants resulted from cognitive aspects. Some participants reported they had problems dealing with sudden negative memories coming up during the stabilizing techniques and guided imagery. Some participants said that they found it easier to concentrate on the instructions in the beginning of the group session. The longer the exercise lasted, the more demanding it became to stay focused. Another obstacle for some participants was the feeling of inner tension. One participant reported that he got distracted by physical pain. The same challenges were encountered when the participants practiced individually. The participants reported that they found it hard to concentrate and focus on the techniques. A few participants thought that self-practice resulted in less improvement of the symptoms than practicing the stabilizing techniques and the guided imagery during the group sessions. Two participants even stopped practicing individually because they felt too weak and without energy.
- **Difficulties due to the absence of structure (28 quotes**): This category includes all statements which represent difficulties through self-practice either between the sessions or after the participants’ last session attendance. The participants described that practicing the techniques during the group sessions was more effective than practicing individually. They found it hard to include the techniques in their everyday routines because they could not remember all of the instructions properly, and felt lost without guidance. Some participants mentioned that the absence of other group members made it more challenging to practice the techniques. Interestingly, one participant said that during the group sessions he was engaged in the techniques in such an intense way, that he felt scared to do the techniques on his own.
- **Difficulties resulting from the surroundings (25 quotes):** Statements concerning difficulties due to the current accommodation are summarized here. The participants reported that one of the central factors that made their self-practice so difficult was the general atmosphere in their accommodation. Most of them shared their rooms with other people. Thus, they were distracted or did not feel comfortable to practice in ‘public’ space. One participant stated that the accommodation did not seem like a comfort zone. Furthermore, the participants mentioned many other conditions which disturbed practicing the stabilizing techniques and guided imagery individually: i.e., their asylum application being rejected, overall insecurity regarding the asylum procedure.

## 4. Discussion

The aim of this study was to qualitatively evaluate a group concept for traumatized refugees focusing on stabilizing techniques and guided imagery. The presented qualitative data enriches our quantitative research on the group therapy sessions focusing on stabilizing techniques and guided imagery [24], which assesses the feasibility and effectiveness of this respective group concept. By conducting semi-structured interviews with the attending participants, we want to gain a more detailed and personal perspective regarding individual experiences with the stabilizing techniques and guided imagery. In health service research, it is quite common to apply a mixed-methods approach [34]. As different authors promote, qualitative and quantitative research can be seen as complimentary, with the qualitative results adding another perspective to the described intervention [35,36].

According to the participants’ statements, they were motivated to join the group sessions focusing on the stabilizing techniques and guided imagery recommended by psychologists and psychiatrists from the psychosocial walk-in clinic in the PHV [26]. Another reason to participate in the group therapy was that they hoped it would help them to overcome the symptoms of mental distress that they were experiencing. Some of the participants reported that they perceived these symptoms as limiting their everyday lives and giving them the feeling of being ‘abnormal’. Thus, many participants were very eager to learn about different ways of living ‘normally’ with their symptoms. These points are very important, because motivation is considered as one of the key factors for treatment success [35].

According to the interviews, many participants thought that the group therapy sessions were helpful due to other group members being present. In order to prevent personal involvement and re-traumatization, we did not allow the participants to talk about their individual, specific trauma histories within the group. Nevertheless, the participants found it important to be surrounded by other people who suffered from similar symptoms. They felt encouraged by sharing their therapeutic experiences and difficulties with the stabilizing techniques and guided imagery. Interestingly, some participants stated that they missed the presence of the other group members during self-practice. In line with these observations, different studies evaluating the benefits of group therapy for adults with traumatic experiences have found that socialization, personal exchange, de-stigmatization, and cohesiveness can improve mental health [12,13,37,38,39,40]. However, two out of three participants left the group because the other group members made them feel uncomfortable. This dropout rate is in line with other studies on group therapy for PTSD patients. Here, approximately 26% of the patients discontinued the group therapy [41,42].

Methodologically, we structured the individual group therapy sessions in a similar way and used the same instructions in each session. This seemed to provide a stable and reliable frame for the participants. They stated that the verbal instructions given by the therapists had helped them to engage in the techniques in a focused way. It is noteworthy, that many participants found it the most challenging for self-practice that they did not have any verbal instructions. These observations are in line with the results of other studies: It has been shown that the internal structure of group therapy sessions helps traumatized patients to stay focused on the present, while preventing intrusive symptoms and dissociation [38,43]. Except for one participant, the participants stated that the intimate atmosphere in the group session room transported a feeling of safety. An important concept for stabilizing therapeutic work in general and for group therapy with male patients in particular, is the reestablishment of safety [14,38,44].

In general, the participants reported that they found the interactions with the therapists very supportive. In particular, they profited from the short discussion round held at the end of each group session after the group had practiced the stabilizing techniques and the guided imagery together. The therapists structured this discussion round by asking the participants whether they felt any different before or after practicing the stabilizing techniques and guided imagery, whether they had noticed any overall improvements, whether they had experienced any difficulties, etc. These questions invited the participants to reflect on their mental state in a guided and focused way. It is important to note, that the participants explicitly mentioned how open and respectful the therapists behaved towards them. In a review Ackerman et al. [45] summarized some personal attributes a therapist should show, i.e., being trustworthy, honest, warmhearted, and open minded. These characteristics help build a therapeutic alliance, which in turn is important for the overall treatment success [45,46,47].

The group members described that they had noticed improvements—both after practicing the stabilizing techniques and guided imagery in the group and after practicing them individually. They stated that they generally felt less distressed or anxious. In particular, they felt more relaxed and optimistic immediately after the group therapy sessions. This is in line with our psychometric results: The participants rated significantly lower anxiety symptoms in the fourth session compared to the first session. Furthermore, we were able to show that stress levels were significantly reduced between the participants’ first and last group sessions [24]. Both trauma and trauma-related problems are dominated by maladaptive feelings like fear, insecurity, and unpredictability [44]. Therefore, it is important to emphasize that the participants in our sample described feeling good, happy, strong, and safe immediately after the sessions. In addition, the participants emphasized that their overall emotions had changed, and that they felt more optimistic. Furthermore, they mentioned that they felt physically and mentally more relaxed after having practiced the stabilizing techniques and guided imagery. Some participants even fell asleep during the sessions. In the long term, the participants noticed that their sleep had improved—a finding which is also described in other studies [13]. The group concept focusing on stabilizing techniques and guided imagery positively impacts the emotional processing and is considered to be beneficial in the setting of a state registration center.

Both on an immediate level and in the long term, the group members noticed that perceptions and awareness in and about their bodies had improved. They said that the felt a deeper connection with their bodies and they felt free and physically stronger. Increased body awareness can help individuals to cope with stress by feeling grounded in the present [44,48,49]. Before practicing the stabilizing techniques and guided imagery, many group participants reported feeling pain in their bodies. This pain was decreased after the group sessions. This aspect is very important when seen in light of the systematic review by Rometsch-Ogioun et al. [50]: The authors revealed that 20–80% of the patients show a connection between their trauma and feeling pain.

According to the participants’ statements, these stabilizing techniques and guided imagery not only had an impact on emotional well-being and physical awareness, but also on a cognitive level. The participants described that they felt less impaired by recurrent and painful thoughts while practicing the techniques. In the long term, the techniques enabled the group members to forget the past for a while by creating an inner distance form past experiences and hurtful memories.

Prior to the first session, many of the participants complained about obsessive thoughts regarding the past or the future, and family members who were not with them. For our participants, stabilizing techniques and guided imagery in general, and Mindful Breathing in particular, had a similar effect as a commonly-applied therapeutic strategy to stop recurrent and negative thoughts. This is very interesting, because a restriction of negative thoughts and emotions helps to experience positive thoughts and feelings, and enables the patient to find positive coping strategies. A previous study has found out that migration populations are more likely to use distracting and avoiding strategies to deal with negative emotions or thoughts [12].

Additionally, the participants described long-lasting effects on self-efficacy. After having practiced in the group as well as individually, the participants emphasized how these techniques helped them to feel more comfortable when being around other people. They said that they felt more engaged in interactions with their peers and felt more capable of concentrating in these encounters. One participant described that he started feeling curious of his surroundings and had gone on some outings to the city next to the accommodation he was sheltered in. Furthermore, some participants explicitly pointed out that they felt more self-confident and in control of their actions. In accordance with our observations, it is assumed that an increase in self-efficacy also influences the intention for action and resulting actions [51]. The participants’ statements are congruent with the aim of the stabilizing techniques and guided imagery to promote self-healing and the strengthening of individual resources. This is also reflected in valence and dominance measures in the quantitative analysis [24,44].

Regarding the challenges of practicing the stabilizing techniques and guided imagery, the participants stated that the cognitive aspects made it hard for them to engage in the techniques. Both when practicing in the group setting and individually, the participants found it hard to concentrate and focus on the instructions. The participants further expressed that they easily got distracted when experiencing pain, having irritating feelings, or too many thoughts and hurtful memories. This is not surprising, given the fact that traumatic experiences and trauma-specific disorders are characterized by overwhelming and unpredictable symptoms [52]. Therefore, we recommended in the group sessions that the participants practice the techniques on a regular basis (at least two times a week in the group and additionally by themselves). Despite these difficulties, the interviews showed that practicing the techniques several times a week enabled the participants to better deal with their symptoms.

According to the participants’ statements, their self-practice was disturbed most by environmental aspects and post-migratory stress factors. Mostly, the participants reported that their accommodation was noisy, and they had to share the room with other refugees. Hence, there was no private space or comfort zone for them. Moreover, many participants felt that post-migratory stress factors, like appointments at the counseling center or with a lawyer concerning their asylum procedure, had such an impact on their mental well-being that they could not concentrate on practicing the stabilizing techniques and guided imagery individually. As other studies previously pointed out, post-migratory stress factors may lead to an aggravation or chronification of mental health symptoms [6]. Therefore, providing stabilizing interventions is especially important when the overall surroundings of individuals are uncertain.

Finally, we would like to give the reader a brief narrative impression of the therapists’ perspective. The therapists perceived the participants as motivated and grateful for the offer. Despite voicing initial reservations about the stabilizing techniques and guided imagery before the first session, the therapists felt that the majority of the participants were able to get involved with the techniques very quickly and already reported beneficial experiences after the first session. One Nigerian participant kindly described the therapists as ‘magicians’. The therapists felt that the clear structure of the sessions was helpful and reassuring for the participants. Most of participants were able to learn to focus on breathing within the first sessions, with most participants requiring several sessions for experiences related to Body Scan and guided imagery. Furthermore, the more sessions the participant attended, the more they seemed to be able to differentiate self-perceptions with regard to emotions and body sensations.

To introduce guided imagery, the therapists had initially chosen to start with the ‘Tree’ technique. However, experience showed that especially participants from Africa did not associate the inner picture of a tree with the same symbolism, such as strength and steadfastness, as is common in the Western world. For them, a tree was more connected to plantations or small bushes, and did not represent a symbol of strength and security. Consequently, the therapists decided to focus on the ‘Inner safe place’ guided imagery technique.

The therapists also perceived themselves as key contacts with regard to questions about German culture, medication intake, or the German asylum procedure. It was important for the therapists to offer the participants an inviting, warm, and colorful room in contrast to the partly sterile realities of the refugees’ accommodations. Hence, the room was decorated with pictures on the walls; the chairs had cushions and a globe served as a point of orientation and symbol of togetherness. During the cold German winter, the participants were also provided with tea, especially when the heating did not work. Sometimes, the sheer gravity of the participants’ past terrors, current instabilities and unclear future situation caused the therapists to feel overwhelmed and powerless. However, the therapists felt their work to be very fulfilling and the stabilizing and guided imagery techniques to be helpful in the early post migratory phase.

This study has several limitations. First, this study relies on self-report interviews as is common in qualitative research. However, we should consider a possible bias of social desirability. We conducted a total amount of 50 interviews among 30 different participants; 26 follow-up interviews were done via cellphone. As different studies pointed out, a physical distance between interviewer and interviewee can reduce the bias of social desirability [53]. Second, we did not check for heterogeneous age, education, or country distributions when we conducted interviews with the participants. Due to the open group setting and the high redistribution quote for T1, we asked the first 16 participants who attended the group therapy sessions if they would like to take part in an interview. For T2 and follow-up interviews, we did not select the participants on a random basis, and tried to contact as many participants as possible. For the follow-up interviews, we tried up to three times to get into contact with the participants. All in all, we collected 50 interviews. According to Hagaman et al. [33], around 16 interviews provide for saturation of the most common categories, while 20–40 interviews are required for saturation of the main themes in heterogeneous populations. Third, the mother tongue of all participants, interviewers, and therapists was not English. That might have caused difficulties understanding the questions. Fourth, besides the participants’ perspective it would have been interesting and useful to analyze the therapists’ perspective in more detail in order to elucidate facilitating and inhibiting institutional and structural factors during preparation and implementation of the stabilization group sessions. It cannot be ruled out that refugees might have had difficulties with self-refection and self-perception. Therefore, an assessment of the therapists regarding the visible changes and processes of the participants would have been valuable. Fifth, all changes in mental wellbeing may not only be caused by the stabilizing techniques and guided imagery, but may be due to other stabilizing circumstances.

## 5. Conclusions

The qualitative semi-structured interviews revealed that the stabilizing techniques and guided imagery are an appropriate intervention for traumatized refugees living in a state registration center. The open group concept provided a structure for male refugees, who were willing to practice the stabilizing techniques and guided imagery, to reflect on their experiences, and to share their thoughts with the other participants and the therapists. Furthermore, the group setting created the basis for the participants to be able to practice the stabilizing techniques and guided imagery individually, once they were not able to join the group anymore. The main challenge to continue practicing the exercises was the absence of instructions. Therefore, we propose that future research concentrates upon alternative ways of teaching and practicing the stabilizing techniques and the guided imagery, such as providing electronic audio-versions.

## Figures and Tables

**Table 1:** Interview guide for interviews conducted after the first group therapy session (T1), after the fifth group therapy session (T2) and two weeks (follow-up) after the participants’ individual last group therapy session attendance.

Interview guide for the interviews at T1 and T2
How was this training session for you today? Why did you come here for the training session? What was helpful in the training session? What was difficult in the training session? Compare yourself now with before the session. Did this training session impact/affect/ help you in some way?
Interview guide for follow-up Interviews
Why did you not come again to the training session? Do you practice the techniques on your own? Compare yourself now with the first time we met when you came to this group. Did this training session impact/affect/help you in some way? What was difficult in the training session?

**Table 2:** Sociodemographic characteristics for all participants (*n* = 30).

	*n*	*%*	
Region of origin			
Sub-Saharan Africa	23	76.7%	
Middle East	3	10.0%	
South Asia	4	13.3%	
Religion			
Christian	18	60.0%	
Muslim	11	36.7%	
Hindu	1	3.3%	
Status of education			
Pupil	6	20.0%	
Finished school	1	3.3%	
No apprenticeship	7	23.3%	
Finished Apprenticeship	7	23.3%	
Student	6	20.0%	
No information	3	10.0%	
Medication			
None	8	26.7%	
Antidepressants	19	63.3%	
Neuroleptics	3	10.0%	
Year of leaving home country			
Before 2014	8	26.7%	
2014	5	16.7%	
2015	4	13.3%	
2016	6	20.0%	
2017	6	20.0%	
No information:	1	3.3%	
	*M*	*SD*	*Range*
Age	25.83	6.80	18–42
Stay in PHV (weeks)	9.57	8.23	1–32
Session attendance	7.03	8.56	1–40
Quantitative baseline scores			
PC-PTSD-5	3.87	1.17	1–5
PHQ-2	3.53	1.48	1–6
GAD-2	4.2	1.52	1–6

Note: PHV = state registration and reception center for refugees ‘Patrick-Henry Village’ in Heidelberg -Kirchheim, Germany; Session attendance = participation in group therapy sessions focusing on stabilizing techniques and guided imagery. PC-PTSD-5 = Primary Care Posttraumatic Stress Disorder Screen for Diagnostic and Statistical Manual of Mental Disorders 5; PHQ-2 = two-item Patient Health Questionnaire; GAD-2 = short version of the General Anxiety Disoder-7.

**Table 3:** Main themes, their categories and exemplarily codings and quotes derived from qualitative inductive content analyses of the total of 50 interviews with *n* = 30 participants having participated in group therapy sessions focusing on stabilizing techniques and guided imagery.

Categories and Themes	Codings	Quotes
(A) Motivation to attend the first group session	Internal motivation because it could be helpful	*“Because it helps me a lot, so that’s why I come to the group sessions-you know–[…]. It’s helping me to try to put the past behind me. It’s helping me”*
(B) Appraisal of the group setting		
• Other group members being present	Exchange of experiences	*“I think it’s good to have two - three people around, you know. At least we can share our idea how we feel, what we feel, what we imagine and how our body works, you know”*
	Uncertainties regarding the others	*“* *Within the group session, I can say everything but- like - something when I watch it, I prefer to share it with the therapists only* *”*
• Atmosphere of the group	Instructions are helpful	*„* *It’s truly because of the instructions that I really feel somehow good now. I always try to focus on the instruction. And then I can’t feel numb. The instruction really helps*
• Support by the therapists	Support through talking to therapists	*“If you come here, you’re [therapists] just talk with us, in that moment you have to forget about things - you don’t have to think about it.”*
(C) Achievements by practicing stabilizing techniques and guided imagery		
• Effects on an emotional level	Calming	*“The session was helpful because […] before I came here, there were not more than 10 min without having some thoughts about something is coming for me, very frightening. Yeah. [...] But when I come and I exercise, participate, I try to calm, try to calm myself”*
	Perception of feelings	*“If you do this training it is good because I feel. Even when I do it, I feel. Like my body is pulled down, I am becoming normal”.*
• Effects on a physical level	Body perception	*“I feel myself in a different way; I don’t feel the same distrained. So if I come here, I feel a lot different in my body so I’m happy. It is because my body is all is free”*
• Effects on a cognitive level	Forget about the past	*“A difference I feel, because–really-it’s something that I think for the first time, like to imagine something, that is something that will even for a second or a minute makes me forget about the pain of the past […]. So for the first time then at least, even if it’s just for a minute and something, new thoughts are coming and I forget about, about the pain.”*
• Effects on self-efficacy	More confident in social contacts	*“But since I started in this group, sometimes I feel happy when I’m with the people. […]But I see the difference: since I started this group I know how to speak to the people, how to get close to the people. […] When I want to get close to them, I will get close to them, talk to them, to chat, we do something joking. You know”.*
	In control	*“I will be able to control the situation that’s why every time I feel that I’m out of control then I do the techniques again to get me in control and normality. That’s why I do it - to bring my mind back to where I really want it to be”.*
(D) Difficulties in practicing the techniques		
• Symptom-related difficulties	Thoughts are disturbing	*“I imagined something but I was confused because there was a lot of thinking in my mind”*
	Concentration difficulties	*“When I try to do the exercises on my own I don’t concentrate. So, normally, when the therapists did the exercises for me and for the group I felt changes. When I do it on my own I don’t feel that relaxed”.*
• Difficulties due to the absence of structure	No guidance in self-practice	*“I realize that it works more when I’m in the group, guided by someone, than when I’m on my own.”*
• Difficulties resulting from the surroundings	Noises and people are distracting	*„Here is difficult to find such environment, […] people are coming and going out. Noises and so forth, they are distracting you also. Yea. I make sure I do it once a day. Imagining, try to deeply breathe in and out, that helps me a lot“*

Note: The codings and quotes shown here are only examples of our inductive content analysis which should illustrate the formation of categories and themes.
